# Two New Coumarins from *Micromelum falcatum* with Cytotoxicity and Brine Shrimp Larvae Toxicity

**DOI:** 10.3390/molecules17066944

**Published:** 2012-06-06

**Authors:** Xiongming Luo, Weihong He, Hao Yin, Qingxin Li, Qiao Liu, Yongzhong Huang, Si Zhang

**Affiliations:** Key Laboratory of Marine Bio-resourses Sustainable Utilization, South China Sea Institute of Oceanology, Chinese Academy of Sciences, Guangzhou 510301, China; Email: luoxm163@sina.com (X.L.); whhe@scsio.ac.cn (W.H.); yinhao@scsio.ac.cn (H.Y.); liqx81@163.com (Q.L.); lq_11@hotmail.com (Q.L.); tj6188@sina.com (Y.H.)

**Keywords:** *Micromelum falcatum*, coumarins, brine shrimp larvae toxicity, cytotoxicity, antibacterial activity

## Abstract

Two new coumarins, 7-methoxy-8-(2-hydroxmethyl-1-O-isovaleryl-4-butenyl)-coumarin (**1**) and 7-methoxy-8-(1-hydroxy-2-O-β-glucopyranosyl-3-methyl-4-butene-1-yl)coumarin (**2**), and twelve known coumarins **3–14** were isolated from the stem bark of *Micromelum falcatum*. The structures of compounds **1–14** were elucidated by extensive spectroscopic data analyses. The toxicity of compounds **1–14** was tested using a brine shrimp assay and *in vitro* antiproliferative assay against mammary cancer (F10) and lung cancer (HvEvc) cell lines by the MTT method. Some compounds had moderate activities. All compounds were also tested against the microorganisms *Bacillus subtilis*, *Bacillus thuringiensis* and *Escherichia coli*, but no activity was observed.

## 1. Introduction

The genus *Micromelum* (Rutaceae family) contains about 11 species which are distributed in Asian tropical and subtropical regions. Phytochemically, many bioactive compounds, including 6- and 8-shi aprenylated coumarins, polyoxygenated flavonoids, dimeric indole alkaloids and carbazole alkaloids were isolated from this genus [1,2,3,4,5,6,7,8]. *M. falcatum* (Lour.) Tan. is a medicinal plant mainly distributed in southern and southwestern China, and widely used in Chinese folk medicine to treat infected wounds, odynolysis, rheumatism, cough and fevers. Early studies of this species have resulted in the isolation of four alkaloids, ten coumarins and three dihydrocinnamic acid derivatives from its leaves and roots collected from Vietnam and China. Many 6- and 8-prenylated coumarins have been reported showing both *in vitro* and *in vivo* antitumor activity [5,6,7,8,9,10,11]. For instance, micromelin exhibited significant *in vivo* activity in mice against P-388 lymphocytic leukemia (T/C 149% at 10 mg/Kg) and Lewis lung carcinoma (T/C 228% at 1.25 mg/Kg) [9], and murrangatin and minumicrolin were valuable anti-tumor promoting agents [10] while microminutin (**3**) [11] displayed *in vitro* activity (ED_50_ 3.7 μg/mL) in the P-388 lymphocytic leukemia test system.

In order to search for new antitumor agents, we further investigated the chemical constituents of this species. Herein, we report the isolation and structure elucidation of two new coumarins, 7-methoxy-8-(2-hydroxymethyl-1-*O*-isovaleryl-4-butenyl)coumarin (**1**) and 7-methoxy-8-(1-hydroxy-2-O-β-gluco-pyranosyl-3-methyl-4-butene-1-yl)coumarin (**2**), and twelve known coumarins, microminutin (**3**) [11], 6-formyl-7-methoxycoumarin (**4**) [12], murralongin (**5**) [13,14], murraol (**6**) [15], arscotin (**7**) [16], murralonginol (**8**) [17], (*E*)-osthenone (**9**) [18], isomurralonginol (**10**) [13,14], murracarpin (**11**) [16], microminutinin (**12**) [6,19], methoxymicrominutinin (**13**) [6,19] and microfalcatin isovalerate (**14**) [6] from the stem bark of *M. falcatum*. The brine shrimp toxicity and cytotoxicity data of compounds **1–14** are also disclosed. No activity was observed in antibacterial tests of all isolated compounds against the strains *Bacillus subtilis*, *Bacillus thuringiensis* and *Escherichia coli*. 

## 2. Results and Discussion

The stem bark of *M. falcatum* was extracted with ethanol to yield a crude extract. The crude extract was dissolved in H_2_O, extracted first with *n*-hexane and then with EtOAc. The EtOAc extract was separated by sequential chromatography on a normal phase silica gel flash column, Sephadex LH-20 column and reversed phase HPLC to afford two new compounds **1–2** and twelve known compounds **3–14**. The known compounds was identified as microminutin (**3**), 6-formyl-7-methoxycoumarin (**4**), murralongin (**5**), murraol (**6**), arscotin (**7**), murralonginol (**8**), (*E*)-osthenone (**9**), isomurralonginol (**10**), murracarpin (**11**), microminutinin (**12**), methoxymicrominutinin (**13**) and microfalcatin isovalerate (**14**), on the basis of MS, ^1^H- and ^13^C-NMR data analyses and comparisons with relevant literature reports. 

**Figure 1:**
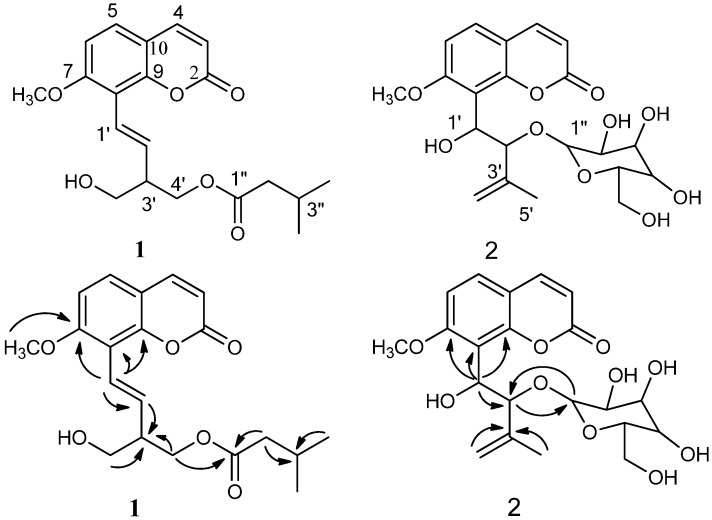
Structures of compounds **1–2** andselected HMBC correlations (H→C) for compounds **1–2**.

Compound **1** was isolated as a colorless oil and had the molecular formula C_20_H_24_O_6_ as determined by HREI-MS (*m/z*: 360.1574 [M]^+^). The UV spectrum of **1** (283.0, 323.3 nm) exhibited the charicteristic absorption bands of a 7-oxygenated coumarin skeleton and the IR data (3540, 1739, 1733, 1605, 1543, 1450, 1224 cm^−1^) showed benzene ring, ester and hydroxy groups. Detailed analyses of the 1D and 2D (COSY, HSQC, and HMBC) NMR spectral data (Table 1) of **1** revealed the presence of an 8-substituted 7-methoxycoumarin moiety, an isovaleryl moiety, and a △^1′^-4′,5′-dioxygenated isoprenyl moiety. The COSY correlations between H-4″ (δ 0.96)/H-3″ (δ 2.12)/H-2″ (δ 2.24) and HMBC correlations observed from H-2″ to C-1″ (δ 173.5), C-3″ (δ 25.7), C-4″ (δ 22.5) and C-5″ (δ 22.5), from H-3″ to C-1″, C-2″ (δ 43.4), C-4″ and C-5″, and from H-4″ to C-2″, C-3″, and C-5″, proved the presence of the isovaleryl moiety. The COSY correlations between H-1′ (δ 6.90)/H-2′ (δ 6.68), H-2′/H-3′ (δ 2.82), H-3′/H-4′ (δ 4.30), and H-3′/H-5′ (δ 3.76), suggested the presence of △^1′^-4′, 5′-dioxygenated isoprenyl unit, which was further supported by the HMBC correlations between H-1′/C-2′/C-3′, H-2′/C-3′/C-4′, H-3′/C-2′/C-4′, H-4′/C-2′/C-3′/C-5′, and H-5′/C-2′/C-3′/C-4′. The three moieties were readily assembled through a C-1′′/C-4′ ester linkage and a C-1′/C-8 carbon-carbon bond, on the basis of the HMBC correlations observed between H-4′/C-1″ (δ 173.5) and H-1′/C-7/C-8/C-9, repectively. The 5′-hydroxy group was deduced from the 1D NMR and ESI-MS data. The HMBC correlation between δ_H_ 3.93 (3H, s) and C-7 (δ 160.2) located the methoxyl group at C-7. The 16.4 Hz coupling constant between H-1′ and H-2′, indicated that the double bond at C-1′ and C-2′ was *trans*. Hence, compound **1** was identified as 7-methoxy-8-(2-hydroxmethyl-1-O-isovaleryl-4-butenyl)-coumarin (Figure 1).

**Table 1:** ^1^H NMR (500 Hz) and ^13^C-NMR (125 MHz) Data for Compounds **1** and **2** ^a^.

	1 (DMSO-d_6_)			2 (MeOD)		
	^1^H-NMR	^13^C-NMR	HMBC	^1^H-NMR	^13^C-NMR	HMBC
2		160.8			162.8	
3	6.26 (1H, d, 9.5 Hz)	113.2	C-2, 4	6.26 (1H, d, 9.5 Hz)	113.3	C-2, 4, 10
4	7.62 (1H,d, 9.5 Hz)	143.8	C-2, 3, 5, 10	7.89 (1H, d, 9.5 Hz)	146.3	C-2, 5, 7, 9, 10
5	7.31 (1H, d, 8.7 Hz)	127.2	C-6, 7, 10	7.58 (1H, d, 8.5 Hz)	130.7	C-4, 7, 9
6	6.86 (1H, d, 8.7 Hz)	107.6	C-5, 7, 8, 9	7.06 (1H, d, 8.5 Hz)	109.7	C-7, 8, 10
7		160.2			162.5	
8		113.7			117.2	
9		153.0			152.8	
10		113.0			114.5	
1′	6.90 (1H, d, 16.4 Hz)	121.6	C-7, 8, 9, 2′	5.57 (1H, d, 9.0 Hz)	68.1	C-7, 8, 9, 2′
2′	6.68 (1H, dd, 16.4, 8.6 Hz)	134.1	C-1′, 3′, 5′	5.18 (1H, d, 9.0 Hz)	85.2	C-1′, 3′, 1′′
3′	2.82 (1H, 6.4, 6.8, 8.6 Hz)	46.7	C-2′, 4′, 5′		143.1	
4′	4.30 (2H, dd, 6.8, 11.5 Hz)	64.1	C-2′, 3′, 1′′	4.70, 4.79 (each 1H, s)	117.0	C-2′, 3′
5′	3.76 (2H, dd, 6.4, 11.0 Hz)	63.0	C-3′, 4′	1.64 (3H, s)	17.3	C-2′, 3′, 4′
1″		173.5		4.28 (1H,m)	100.9	2′, 2′′
2″	2.24 (2H, d, 7.1 Hz)	43.4	C-1′′, 3′′, 4′′, 5′′	3.37 (1H, m)	74.9	1′′, 3′′
3″	2.12 (1H, 6.6, 7.1 Hz)	25.7	C-1′′, 2′′, 4′′, 5′′	3.37 (1H, m)	78.1	2′′, 4′′
4″	0.96 (3H, d, 6.6 Hz)	22.5	C-,2′′, 3′′5′′	3.37 (1H, m)	71.8	3′′, 5′′
5″	0.96 (3H, d, 6.6 Hz)	22.5	C-,2′′, 3′′5′′	3.27 (1H, m)	77.8	4′′, 6′′
6″				3.73, 3.92 (each 1H, m)	62.8	5′′
CH_3_O	3.93 (s)	56.1	C- 7	3.97 (3H, s)	56.7	C-7

^a^ Assignments made on the basis of HSQC and HMBC.

Compound **2** was isolated as a colorless oil and was found to have the molecular formula C_21_H_26_O_10_ as established by HR-EIMS (*m/z*: 438.1528 [M]^+^). Careful examination of the NMR spectra of **2** (Table 1) implied that **2** contained an 8-substituted 7-methoxy coumarin moiety, a prenylated group and an glucose moiety. The HMBC correlations between H-1′ (δ 5.57)/C-2′ (δ 85.2), H-2′ (δ 5.18)/C-1′ (δ 68.1)/C-4′ (δ 117.0), H-4′ (δ 4.70, 4.79)/C-2′/C-3′(δ 143.1)/C-5′(δ 17.3) and H-5′ (δ 1.64)/C-2′/C-3′/C-4′established the prenylated carbon sequence, which connected with C-8 of the 8-substituted 7-methoxycoumarin moiety at C-1′ by the HMBC signals between H-1′ (δ 5.57)/C-7 (162.5)/C-8 (117.2)/C-9 (152.8). Based on the HMBC correlation from δ_H_ 3.97 (3H, s) to C-7 (δ 162.5), a methoxy group was placed at C-7. A glucose group was located at C-2′ as deduced from the COSY correlations between H-1′′/H-2′′, H-2′′/H-3′′, H-3′′/H-4′′, H-4′′/H-5′′, H-5′′/H-6′′and the HMBC correlations between H-1′′ (δ 4.28)/C-2′(δ 85.2), and H-2′ (δ 5.18)/C-1′′(δ 100.9). Accordingly, the structure of **2** was determined as 7-methoxy-8-(1-hydroxy-2-O-β-glucopyranosyl-3-methyl-4-butene-1-yl)coumarin (Figure 1).

Toxic activities of compounds isolated from *M. falcatum* were tested against brine shrimp larvae using a 96 well plates assay and among all compounds tested, compound **1** had strong activity (LC_50_ < 10 μM), and exhibited an LC_50_ value of 6.8 μM, indicating that **1** was a potent toxic natural product. Compounds **2**, **3**, **6**, **7**, **10**, **11**, and **14** had moderate activity (LC_50_ < 500 *μ*M), and the LD_50_ values of all isolated compounds are displayed clearly in Table 2. 

Compounds **1–14** were further evaluated for their *in vitro* antiproliferative activities against mammary cancer (F10) and lung cancer (HvEvc) cell lines by the MTT method, and compounds **2**, **3**, and **14** displayed moderate activity against the F10 cell line with IC_50_ values of 23.6, 82.9 and 112.0 μg/mL, respectively, and compounds **1**, **2**, **6**, **10** and **11** displayed moderate activity against the HvEvc cell line with IC_50_ values of 35.7, 68.5, 172.5, 72.6 and 124.3 μg/mL, respectively. 

**Table 2:** The LD_50_ values of compounds **1–14** against brine shrimp larvae and the IC_50_ values of all compounds against mammary cancer (F10) and lung cancer (HvEvc) cell lines.

Compound	LD_50_ (brine shrimp) μg/mL	IC_50_ (F10) μg/mL	IC_50_ (HvEvc) μg/mL
**1**	6.8	—	35.7
**2**	29.1	23.6	68.5
**3**	107.3	82.9	—
**4**	>500	—	—
**5**	>500	—	—
**6**	199.3	—	172.5
**7**	118.5	—	—
**8**	>500	—	—
**9**	>500	—	—
**10**	50.6	—	72.6
**11**	158.5	—	124.3
**12**	>500	—	—
**13**	>500	—	—
**14**	257.4	112.0	—

In addition, no antibacterial activity was observed in the test of all compounds against *Bacillus subtilis* (SCSIO00189), *Bacillus thuringiensis* (SCSIO00190) and *Escherichia coli* (SCSIO00191) by the K-B disk diffusion method.

## 3. Experimental

### 3.1. General

Optical rotation was measured on Polaptronic-HNQW5 high-resolution polarimeter. NMR spectra were recorded on a Bruker DRX-500 spectrometer with SiMe_4_ as internal standard. ESI-MS was measured with a API2000 LC/MS/MS mass spectrometer (Applied Biosystems). HREI-MS was recorded on a Thermo MAT95XP spectrophotometer. Silica gel (200–300 mesh, Qingdao Haiyang Chemical Plant, Qingdao, China) and Sephadex LH-20 (Pharmacia) were used for column chromatography. Thin layer chromatography (TLC) was carried out on precoated silica gel G plates (Qingdao Haiyang Chemical Plant, Qingdao, China) and spots were visualized by spraying the plates with 50% H_2_SO_4_ solution, followed by heating. Semi-preparative RPHPLC was carried out on ODS columns (YMC-Pack ODS-5-A, 250 × 10 mm, 5 μm, YMC) with the CH_3_OH–H_2_O solvent system as eluents. A Waters 600 HPLC system equipped with a Waters 996 photodiode array detector was used for HPLC analysis.

### 3.2. Plant Material

The stem bark of *M. falcatum* (Lour.) Tan. was collected in October 2007 in Sanya, Hainan Province, China, and identified by Prof. Si Zhang. A voucher specimen is deposited at the Herbarium of South China Sea Institute of Oceanology (accession number: Dajian 020). 

### 3.3. Extraction and Isolation

The air-dried material *M. falcatum* (Lour.) Tan. (5.0 kg) was extracted with 95% EtOH (50 L) three times, respectively. The organic solvent was combined and evaporated under reduced pressure to give a residue. The residue was disolved in 2L H_2_O and was extracted sucessively with *n*-hexane and EtOAc (2 L, each 4×) to yield 38 g *n*-hexane extract and 76 g EtOAc extract. The EtOAc extract was separated on silica gel (820 g, 200–300 mesh) with solvents of increasing polarity: 10–70% acetone in *n*-hexane followed by 5–100% MeOH in CHCl_3_ to afford 102 fractions. Frs. 15–18 (2.15 g, eluted with *n*-hexane-acetone 7:3) was combined and again chromatographed on silica gel using chloroform-acetone (20:1) to afford compound **12** (135 mg). Frs. 20–21 (2.38 g, eluted with *n*-hexane-acetone 65:35) was chromatographed on silica gel with chloroform–acetone (15:1) and afforded frs. 20a–c. Fr. 20a was further purified by chromatography on Sephadex LH-20 with MeOH to afford compound **4** (6.8 mg), **5** (15.7 mg), **6** (12.2 mg), **8** (5.4 mg), and **13** (24.5 mg). Frs. 39–42 (2.05 g, eluted with *n*-hexane–acetone 6:4) was fractionated on silica gel using chloroform–acetone (8:2) to give compound **1** (4.8 mg), **7** (7.7 mg), **9** (14.2 mg), **10** (21.4 mg) and **11** (11.8 mg) which was crystallized from MeOH. Frs. 45–48 (2.60 g, eluted with *n*-hexane-acetone 6:4) was fractionated on silica gel with chloroform-acetone (8:2) and then purified by semi-preparative HPLC using MeOH/H_2_O as eluents (from 35:65 to 55:45) to yield compounds **2** (8.5 mg), **3** (6.1 mg), and **14** (10.6 mg).

*7-Methoxy-8-(2-hydroxmethyl-1-O-isovaleryl-4-butenyl)coumarin* (**1**): colorless oil; [α]^20^_D_ −12.4° (CH_3_OH, c 0.33); UV (MeOH) λ_max_ (logε) 283.0 (3.93), 323.3 (4.22) nm; IR (KBr) ν_max_ cm^−1^ 3540, 1739, 1733, 1605, 1543, 1450, 1224; ^1^H- and ^13^C-NMR data, see Table 1; Positive ESI-MS *m/z* (rel.int.): 743 [2M+Na]^+^ (92), 383 [M+Na]^+^ (100), 361 [M+H]^+^ (68), 316 (35); HR-EIMS *m/z* 360.1574 (calcd for C_20_H_24_O_6_^+^ [M]^+^, at *m/z* 360.1573). 

*7-Methoxy-8-(1-hydroxy-2-O-β-glucopyranosyl-3-methyl-4-butene-1-yl)coumarin* (**2**): colorless oil; [α]^20^_D_ +29.5° (CH_3_OH, c 0.74); UV (MeOH) λ_max_ (logε) 218.2 (1.60), 246.4 (0.90), 323.0 (1.80) nm; IR (KBr) ν_max_ cm^−1^ 3542, 1730, 1607, 1548, 1450, 1220, 1062; ^1^H- and ^13^C-NMR data, see Table 1; Positive ESI-MS *m/z* (rel.int.): 899 [2M+Na]^+^ (70), 461 [M+Na]^+^ (100), 439 [M+H]^+^ (68); HR-EIMS *m/z* 438.1528 (calcd for C_21_H_26_O_10_^+^ [M]^+^, at *m/z* 438.1526). 

### 3.4. The Brine Shrimp Larvae Lethality Bioassay

According to the method described by Wanyoike [20], brine shrimp eggs (Ocean Star International, Inc., USA) were hatched in a large beaker containing natural sea water (South China Sea) and they were cultured at room temperature for 48 h. With the help of a light source, the larvae grouped together on one side of the vessel and were easily collected for the assay. The compounds **1–14** were dissolved in dimethyl sulfoxide (DMSO) at the concentration of 50 mg/mL, and then diluted in 96 well plate with 200 µL sea water for testing at the final concentrations of 5, 50 and 500 μg/mL. Each test was processed in triplicate with approximate ten larvae. Brine shrimps were counted under a magnifying glass after 24 h of incubation and maintaining the 96 well plates under illumination. The controls were prepared in the same manner except that the test samples were omitted. The lethality of dead larvae was recorded and used for calculating the LC_50_ by the Lanyu LC_50_ analysis program (Version 1.01).

### 3.5. Antiproliferative Assays

Antiproliferative activities of compounds were evaluated by the MTT method using mammary cancer (F10) and lung cancer (HvEvc) cell lines. In MTT assay, the cell suspensions (200 μL) at a density of 1 × 10^5^ cells mL^−1^ were plated in 96-well microtiter plates and incubated for 24 h at 37 °C in a humidified incubator at 5% CO_2_. The tested compound solution (2 μL in DMSO) at different concentrations was added to each well and further incubated for 72 h in the same condition. Then, the MTT solution (50 μL) was added to each well and incubated for 4 h. The old medium (150 μL) containing MTT was then gently replaced by DMSO. Absorbance was then determined on a Spectra Max Plus plate reader at 490 nm. 

### 3.6. Antibacterial Assay

Compounds were tested against the microorganisms *Bacillus subtilis*, *Bacillus thuringiensis*, *Escherichia coli* and rifampicin was used as positive control for the three microorganisms. Procedures for the antimicrobial susceptibility assays were performed using a modified method [21]. Compoundswere dissolved in absolute DMSO to give the needed amounts of 0.1, 1.0, and 10 μg per 6 mm diameter paper disk, respectively. Twelve cm diameter dishes, filled with LB medium, were set for each microbe species. The inhibition zones surrounding each filter paper disk were measured at the end of an incubation period of 24 or 48 h at 27 °C. The absolute DMSO alone showed no inhibition zone (control).

## 4. Conclusions

Bibliographical research revealed some information about the chemistry of four species of *Micromelum; Micromelum falcatum, M. minutum, M. integerrimum and M. zeylanicum* and that many significant 6-prenylcoumarins and 8-prenylcoumarins metabolites were isolated from them. We now report the isolation and structural elucidation of 14 coumarins including 6-prenylcoumarins [6-formyl-7-methoxycoumarin (**4**), murraol (**6**), arscotin (**7**), methoxymicrominutinin (**13**)], and 8-prenylcoumarins [7-methoxy-8-(2-hydroxmethyl-1-O-isovaleryl-4-butenyl)coumarin (**1**), 7-methoxy-8-(1-hydroxy-2-O-β-glucopyranosyl-3-methyl-4-butene-1-yl)coumarin (**2**), microminutin (**3**), murralongin (**5**), murralonginol (**8**), (*E*)-osthenone (**9**), isomurralonginol (**10**), murracarpin (**11**), microminutinin (**12**), and microfalcatin isovalerate (**14**)]. From the literature prior to this study and this paper on genus *Micromelum*, the 6-prenylcoumarins and 8-prenylcoumarins as products of metabolism of the taxon can be attributed as indicators of chemotaxonomic significance. Compound **2**, as a new coumarin glycoside, is the first glycoside reported from the genus *Micromelum*. The toxicity of compounds **1–14** was tested by a brine shrimp assay and *in vitro* antiproliferative assay against mammary cancer (F10) and lung cancer (HvEvc) cell lines by a MTT assay. No antibacterial activity of any of compounds was observed against the microorganisms *Bacillus subtilis*, *Bacillus thuringiensis* and *Escherichia coli*. 
